# Correction: A new model for simultaneous dimensionality reduction and time-varying functional connectivity estimation

**DOI:** 10.1371/journal.pcbi.1009112

**Published:** 2021-06-08

**Authors:** The PLOS Computational Biology Staff

The images for Figs 2 and 3 are incorrectly switched. The image that appears as Fig 2 should be Fig 3, and the image that appears as Fig 3 should be Fig 2. The figure captions appear in the correct order. The publisher apologizes for the error.

**Fig 2:**
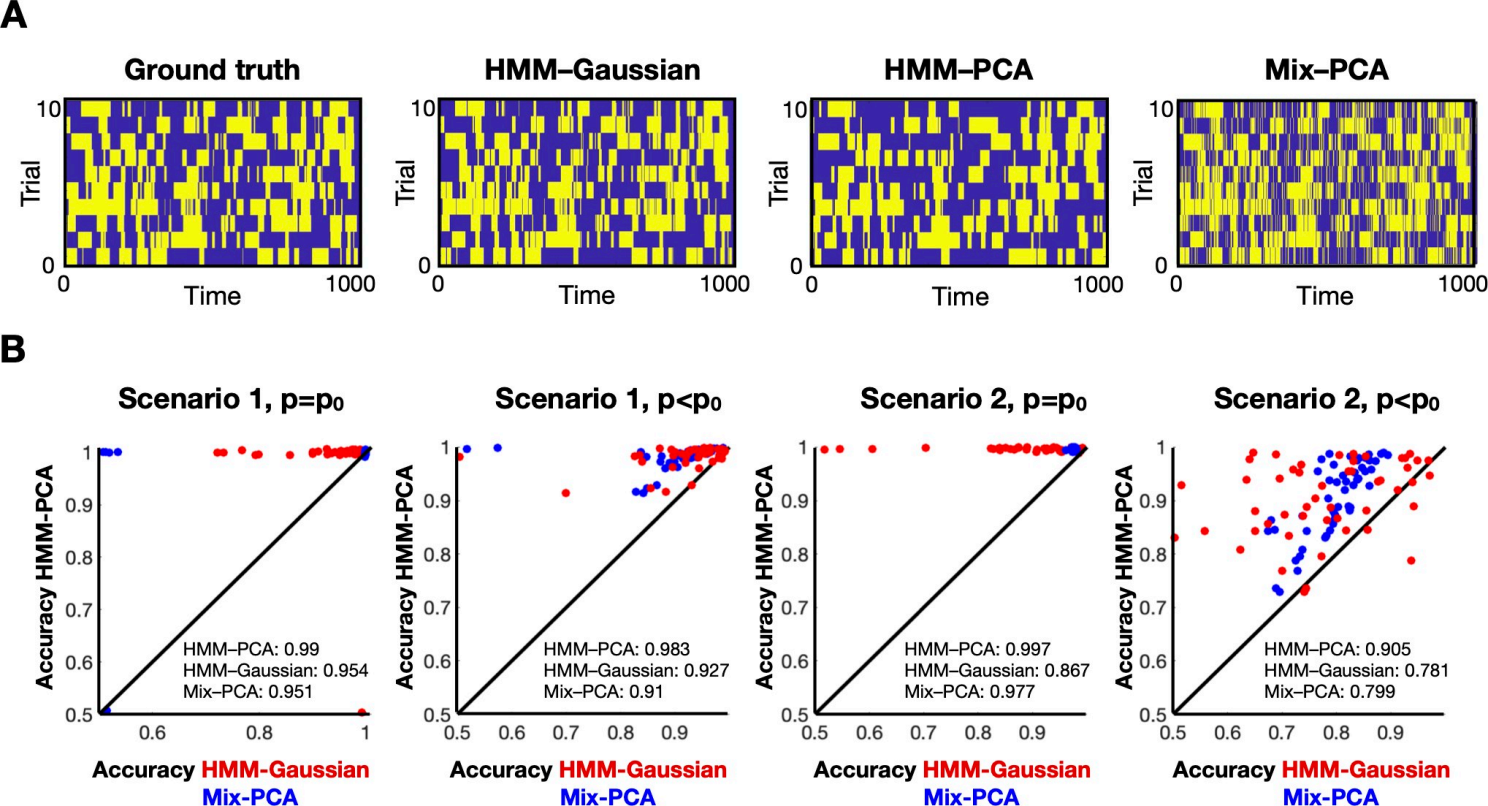
HMM-PCA outperforms the HMM-Gaussian and Mix-PCA approaches on synthetic data. **A**. Example of how the different models recover the ground-truth state time courses. **B**. Comparative accuracy between HMM-PCA (Y-axis), HMM-Gaussian (X-axis, red) and Mix-PCA (X-axis, blue). Each dot represents one repetition of the simulations. Accuracy is measured in terms of how well each method recovered the ground-truth state time courses. Permutation-based statistical testing revealed that HMM-PCA was always significantly more accurate than the other approaches (p<0.001).

**Fig 3:**
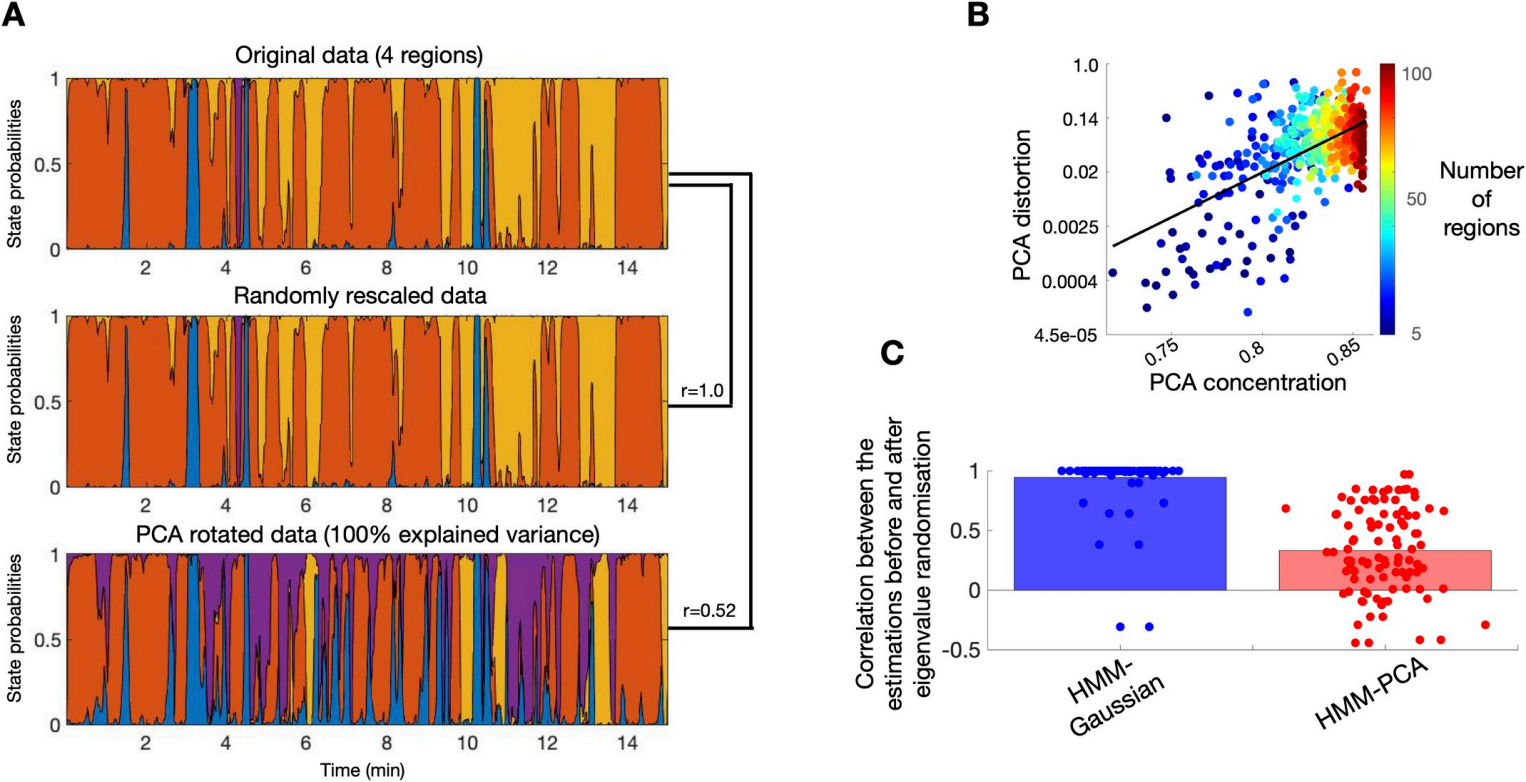
PCA introduces an estimation bias on the HMM or mixture model. **A**. The HMM inference is scale-invariant, but not PCA-rotation-invariant. State time courses produced by the HMM inference for one HCP subject on the original parcellation space (top), after applying a random scaling of the data (middle), and after PCA rotation with no loss of variance (bottom). Each colour represents a different state, so that the coloured areas indicate the probability of activation for the states across the session. The similarity between the different runs, expressed as Pearson’s correlation coefficients, are expressed on the right. **B**. The extent of this bias (PCA distortion) is logarithmically related to how concentrated is the variance on the first PCs (PCA concentration); that is, the more correlated are the regions on the original data, the stronger will be the bias introduced by PCA. **C**. Random manipulations of the data eigenvalues are correctly reflected as changes in the HMM-PCA estimations; HMM-Gaussian is not able to capture the changes.
