# Supplementary figures and images for: A new model for simultaneous dimensionality reduction and time-varying functional connectivity estimation
Source: PLoS Comput Biol. 2021 Apr 16;17(4):e1008580. doi: 10.1371/journal.pcbi.1008580 (PMC8081334; doi:10.1371/journal.pcbi.1008580)

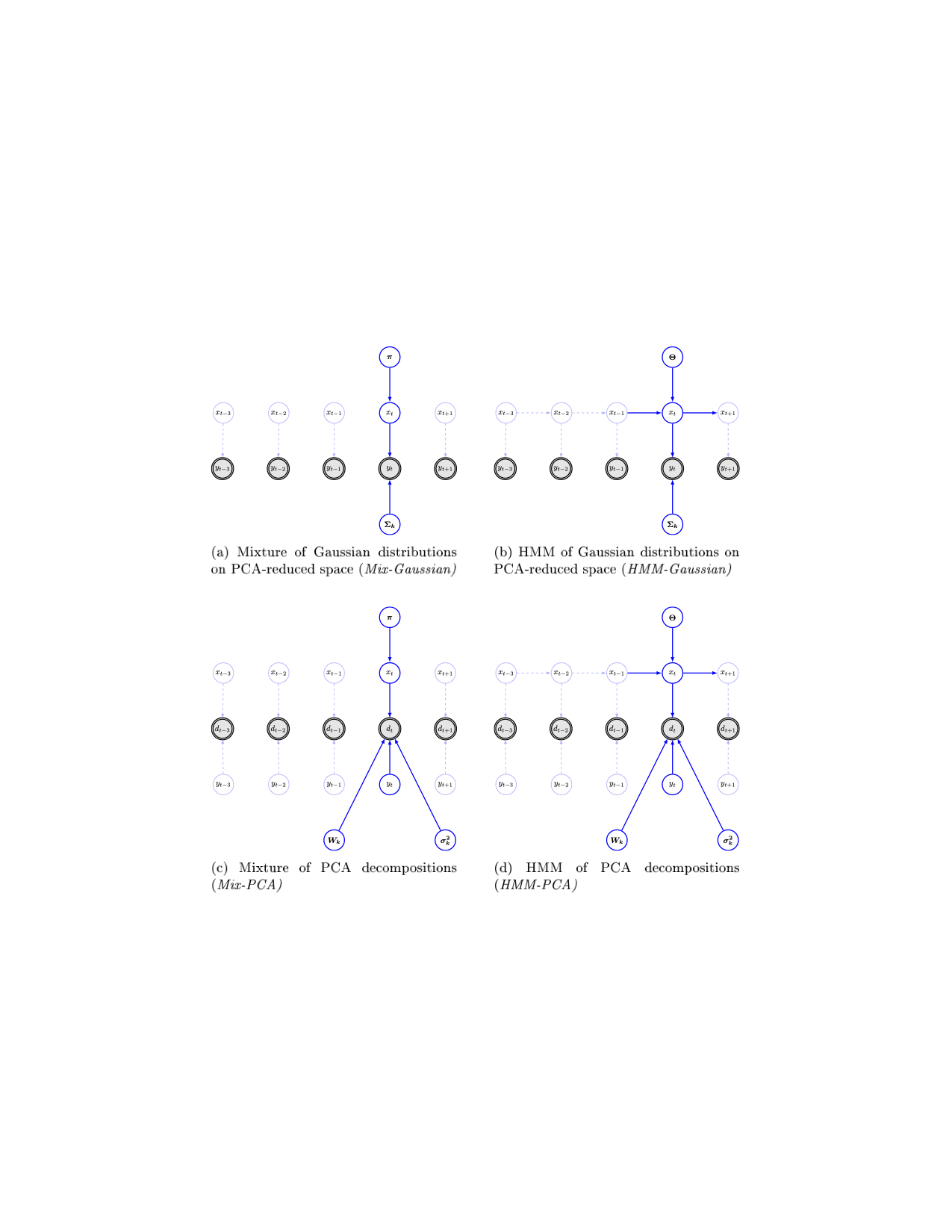

Supplement: S1 Fig — (TIFF) [file pcbi.1008580.s001.tiff]
