# Supplementary material for: A new model for simultaneous dimensionality reduction and time-varying functional connectivity estimation
Source: PLoS Comput Biol. 2021 Apr 16;17(4):e1008580. doi: 10.1371/journal.pcbi.1008580 (PMC8081334; doi:10.1371/journal.pcbi.1008580)
Supplement: Attachment — Submitted filename: ResponseLetter.docx [file pcbi.1008580.s002.docx]

I appreciate the comments from all the Reviewers, which I feel have strengthened the paper considerably. The main changes in the new version of the paper are: (i) I have added a new set of experiments with higher-dimensional data; (ii) I have included comparisons in terms of the free energy (an approximation of the model evidence); (iii) I have clarified the explanation of the HMM in general and of the HMM-PCA in particular to make it more accessible; (iv) I have expanded the Discussion; (v) I have added links to the code. Please find the changes highlighted in red.

**Reviewer #1**

*In this paper, the author proposes an HMM-PCA model to explain time-varying FC in resting state data. The idea is very nice and potentially very useful, but the methodology and results do not adequately support the claims. The paper needs to be improved.*

I thank the Reviewer for both the positive comments and the useful feedback.

*General feedback:
To motivate this approach, I think the author needs to clearly show the generative model (in form of a graphical model) and also report the model evidence, when performing model inversion. As for the inference technique, if one commits to maximum likelihood, then a grid search over the number of HMM hidden states and the number of PCA latent states is essential. Alternatively, if one turns to Bayesian PCA (reference 19), then Bishop's hyperparameter approach will determine the effective dimensionality of PCA, but the optimal cardinality of HMM’s latent space would still rely on grid search. Either way, the model evidence (accuracy minus complexity) will determine whether this new model is superior to HMM+PCA (in 2 steps) or Gaussian mixture model, in the present application. Ideally, this superiority needs to be demonstrated on high-dimensional (simulated) data first, because the claims pertain to the curse of dimensionality. In short, face validation of a new model is conventionally established based on model comparison (in terms of model evidence) and accuracy of the posteriors (with respect to the ground truth). If there is any such thing as bias/distortion or loss of sensitivity (as the author suggests) due to the two-step procedure, these will also show up in the results, alongside the more important model evidence.*

Thanks. I respond to all of these issues in the detailed inline responses below.

*Speaking of the issue of high dimensional data, please note that 50-100 regions/ICs is not really high dimensional when the time series are concatenated across subjects. Moreover, your simulations are low-dimensional (with 10 regions/ICs). So you are not actually tackling the high-dimensional scenario (of many voxels and few time points) neither in the simulations nor in empirical data, to show the reader the gravity of this problem and how you have solved it.*

I agree with Reviewer that the examples in the manuscript are not what is normally considered high-dimensional, although the original fMRI space (around 90K voxels) is high-dimensional. Most importantly, I would like to note that the biases that constitute the main point of this paper are not inherent to the data being high-dimensional, but to the solution against high-dimensionality that is commonly used in the field (PCA). The point of the simulations is to demonstrate the issues of PCA when combined with the HMM or Gaussian Mixture for the purposes of estimating time-varying FC. These could happen with any dimensionality. The fact that the examples are low-dimensional is purely a practical choice, and the conclusions apply generally. Of course, the two issues described in Subsection 2.3.1 and Subsection 2.3.2 also apply to a properly high-dimensional scenario. I have now commented on this in the Discussion:

“Importantly, the cause of the reported biases does not inherently lie on the high dimensionality of the data, but on the use of PCA as a way to reduce such dimensionality. Therefore, although for practical reasons the examples and simulations used throughout the paper are not high-dimensional by normal standards [35], the issues addressed by these examples apply generally.”

[35] P. Buhlmann and S. Van De Geer (2011). Statistics for high-dimensional data: methods, theory and applications. Springer-Verlag Berlin Heidelberg

*Page 2, 2nd paragraph: this criticism to the sliding window analysis (that mere fluctuations may be mistaken for non-stationarity in the connectivity dynamics) applies to the hidden Markov model as well, unless model evidence rules out the existence of only one underlying connectivity state.*

As established in previous work and summarised by Lurie et al (2020), the HMM is able to effectively address the statistical issue of sliding-window analyses (described in Hindriks et al, 2016); that is, that within a sliding-window (eg 1min=80TRs), the amount of data might be insufficient to provide a reliable estimation, so that the variance of the estimation supersedes the actual FC variability in the data. However, within an HMM state the amount of data is orders of magnitude larger (in the example 68.3hours on average per state = 328K TRs), which makes the variance of the estimation negligible. Therefore, if there is a difference of FC between two states, such difference exists in the data. Note that the problem of statistical variability that I discuss here is a different one than the question of whether the data supports one state or more than one (see below about that specific question), and also note that the sliding-window approach is not a generative model and therefore it cannot be assesed through the model evidence.

I have clarified this point in the second paragraph of the Introduction as follows:

“For example, if an HMM with twelve states was trained on 820 subjects from the Human Connectome Project (HCP) data set [17], each state would be on average estimated on 68.3h of data; as opposed to a typical 1min window, the statistical noise in this estimation is negligible.”

*Page 3, 1st paragraph: Please rephrase. Using ambiguous statements such as "works reasonably well in practice for many (but not all) fMRI data" without bringing in quantitative support or a credible reference is not appropriate. Or, for instance, saying that a method "is not designed for the final goal of the analysis" without specifying that final goal, is not precise enough for a scientific text.*

I agree with the Reviewer that these statements are somewhat ambiguous. I have changed the latter one to “designed to the specific goal of quantifying within-session FC modulations”.

With regard to the former statement, this is very hard to quantify meta-analytically because this type of negative result (e.g. trying the HMM and not working) is rarely reported in the literature. However, we are currently working on a piece of work aimed at quantifying just that, and we expect it to be preprinted in the next one or two months. The summary of this work is that this depends on a trade-off between the amount of data, the dimensionality, the amount of parameters in the model and the preprocessing strategy (in particular if Multimodal Surface Matching [38] was performed or not). Whereas this work is not yet ready, I think this sentence (however vague) is still useful for the readers with a neuroimaging background, who often try to apply these methods and, when faced with these issues, doubt if it is a problem of the data or of their pipeline. To make it clearer, it have rephrased it to:

“Whereas this two-step method works reasonably well in practice for many (but not all) fMRI data sets, in the sense that it captures within-session FC modulations at least to some extent, having PCA and the HMM estimation as two separate steps is suboptimal.”

[38] Robinson EC, Jbabdi S, Glasser MF, Andersson J, Burgess GC, Harms MP, Smith SM, Van Essen DC, Jenkinson M. Msm: A new flexible framework for multimodal surface matching. NeuroImage. 2014;100:414–426.

*Page 3, 2nd paragraph: I can't see why the FC matrices are being interpreted as instantaneous, when in practice they are the covariance matrices of the Gaussian distributed observations (of each state).*

I apologise for the lack of clarity here. This sentence is merely enunciating the goal of the analysis, regardless of chosen approach. I have now added the following sentence:

“In either case, an instantaneous FC matrix at time point $t$ can be defined as a linear combination of the state covariance matrices using the state probabilities $\gamma_{tk}$ as weights.”

*Page 3, 4th paragraph: principal components are by design orthogonal. I don't see your point about transient departure from orthogonality. Please elaborate if this is actually what you meant. temporarily*

I have expanded the explanation now as:

“However, there might be periods in the data during which the time series are temporarily not orthogonal, meaning that the time series are temporarily correlated (or negatively correlated) across brain regions. Any transient departure from (the time-averaged) orthogonality, encoded by the HMM parameters $\gamma_{tk}$ and $\boldsymbol\Sigma^{(k)}$, can be considered an FC modulation.”

P*age 3, section 2.2: when a new probabilistic model is proposed, it is common to show the (generative) model structure as a graphical model, clearly showing the conditional (in)dependencies, and the model parameters and hyperparameters.*

I now present the graphical models for the four considered approaches (HMM-PCA, HMM-Gaussian, Mix-PCA and Mix-Gaussian) in the Appendix, Figure 1. This is referred to in the main text in page 4 and on the caption of Figure 1.

*Page 4, 1st paragraph: It seems that you are using probabilistic PCA, not Bayesian PCA (with prior over W), as per your reference 19 (Bishop, Bayesian PCA).*

Thanks. The Reviewer is right, the model corresponds to Probabilistic PCA. I have changed it in the text accordingly.

*Page 4, below Eq.1: I think discounting the mean needs further motivation. Does it have computational or practical advantages? More importantly, do you get higher model evidence by fixing all the means at a prior value of zero?*

This choice is merely motivated by interpretation, not by computational reasons. That is, we wish to find changes in correlation above and beyond changes in amplitude. While supressing the mean does not guarantee that changes in amplitude will not participate in the state estimation, pinning the mean to zero promotes the importance of FC in the HMM estimation [8]. Please note that this type of considerations, driven by the scientific question and by interpretation purposes, are not contemplated by the model evidence (see below for further discussion about this point). I have rephrased the text as:

”Since we are not interested in modelling transient changes in amplitude and we wish to concentrate the model’s explanatory power on FC modulations as much as possible, …”

[8] D. Vidaurre, A.L Arenas, S.M. Smith, and M.W. Woolrich. Behavioural relevance of spontaneous, transient brain network interactions in fmri. *NeuroImage*, In press, 2021.

*Page 4, middle of page: "Note that the estimation of u_tk only depends on the data at time point t and on pi_k, ignoring the data temporal structure". It is true that temporal order is not accounted for in mixture models; however the responsibilities depend on all the data points through the model parameters. So each u_tk does not depend *only on time point t and on pi_k*. Please rephrase to avoid misunderstanding.*

I thank the Reviewer for pointing this out – the expression was not completely correct. I have now rephrased as:

“Note that $\boldsymbol{x}_t$ is conditionally independent of all data time points except $\boldsymbol{d}_t$, therefore ignoring the data temporal structure”

*Page 4, middle of page: "In the HMM-PCA case, we instead have the state latent variables xk modelled as an order-1 Markovian process...". Please draw the generative model to show how the HMM states map to the PPCA states, which are then combined with W to generate the data dt. Also please make sure the reader realizes the difference between these two types of latent states; i.e. the (categorical) hidden states of HMM, as opposed to the (Gaussian) latent states of PPCA. It is useful to stress their dimensionalities as well*

As mentioned above, I have included the graphical models in the Appendix for clarity. I have also explicitly mentioned the dimensionality of the variables now.

*Page 4, Eq.3: Complete data log-likelihood (LHS) is also a function of the model parameters.*

Thanks. I have amended this now.

*Page 4, Eq.4: this is not the familiar form of Bishop's PPCA. If it comes from another source, please provide the reference. Otherwise please put your derivation in the appendix.*

I have not derived these Equations independently. These were taken from Tipping and Bishop (1999), Appendix C. The reference is correct now.

*Page 6: This whole bias/distortion story of PCA (assuming it is serious) has not been demonstrated well. Note that in Fig 2B most of the computed distortion is below 2%. Plus, how often does the first PC become so dominant in a FC pattern to take up 86% of the variance? I think to motivate your approach, you can compare model evidence of HMM+PCA (as a two-step procedure) to your proposed HMM-PCA. Even Fig 2A is not convincing, because there is no ground truth here, so we can not say PCA has caused any distortion. HMM+PCA is simply suggesting a different number (and sequence) of hidden states for the HMM, which may actually be more plausible than the results on the original (unrotated) data, in terms of model evidence. Note that you have not shown model comparison results for the number of hidden states, neither on the original data, nor on the PCA-rotated data. So I think this exemplar plot is not sufficiently supporting the distortion/bias claim.*

There are various interesting points here, and I am grateful to the Reviewer for bringing these up, as they have led to a reconceptualisation of some parts of the Discussion that I believe has enriched the paper significantly.

First, I would like to emphasise that the model evidence is a mechanism to quantify which model is better supported by a given, specific data set. In fact, the point of this section is simply to express that the use of PCA changes the HMM estimation, independently of which specific data set we are using. The model evidence is data-dependent, and therefore is not an appropriate means to demonstrate this general issue.

Apart from the fact that the model evidence is not readily available in this type of inference and that all we have is an approximation (the free energy or lower bound), please note that we cannot straightforwardly claim that a lower free energy implies a biologically better representation, since the HMM is not a realistic biophysical model. As mentioned above, one crucial aspect of these models in the way they are applied in the field is interpretability, which is not considered by the free energy (or model evidence) formulation. For example, increasing the amount of data will lead the free energy to ask for more states, and this might make the solution less interpretable (see below).

A more appropriate argument than the one based on the model evidence is now made in the Discussion: PCA is an artificial (i.e. non-biologically motivated) transformation that we use for convenience. Therefore, if we had infinite data (i.e. if dimensionality or overfitting were not a problem), then we would not want any artificial transformation (e.g. PCA) to have any change over the original estimation. In this sense, the fact that PCA does change the estimation regardless of the amount of data is detrimental per-se.

About Figure 2 being or not a convincing demonstration of the points made in the section, please note that these results are merely an illustration of a mathematical fact. This phenomenon invariably occurs in real data regardless of its dimensionality. This simulation is not intended to quantify how much it actually does; that is, it might well be that real data is at the upper extreme of panel B or even further up. To avoid misunderstandings, I have now added at the beginning of the section:

“Note that the following discussion merely states two mathematical facts. How much these biases actually affect real data will likely depend on many factors including acquisition, preprocessing, and experimental paradigm.”

The Reviewer questions about how often the first PC become so dominant in a FC pattern to take up 86% of the variance is a well-spotted one (but please note that this is not the explained variance of the first component, but the average cumulative explained variance). The answer depends on whether or not global signal regression (Murphy and Fox, 2017) was part of the preprocessing or not. If not, then by the nature of fMRI there will always be a major dominant component in the signal explaining a large amount of variance; if yes, then the PCA concentration will indeed be lower. In the basic HCP data used here, there is no global signal regression. I have briefly mentioned this as:

“For instance, the bias highlighted in Figure 2 would be less severe in data where global signal regression [36] has been performed; otherwise, a single component capturing the global signal could explain a large amount of variance, therefore increasing the PCA concentration measure.”

Also note that Fig 2A is merely indicating that PCA introduces a distortion with respect to the original estimation (upper panel), and not with respect to the ground truth (which as the Reviewer points out, is not available). In that sense, it is clear from the figure that PCA makes the estimation to change. I have clarified this at the beginning of the section:

“The application of PCA systematically alters the HMM or mixture model inference with regard to the original estimation (i.e. the one obtained without using PCA), …”

and at the end of the section:

“(PCA) can also arbitrarily distort the time-varying FC estimates (with respect to a non-PCA estimation) towards the lower-order PCs.”

[36] K. Murphy and M.D. Fox (2017). Towards a consensus regarding global signal regression for resting state functional connectivity MRI. NeuroImage

*Page 6, section 2.3.2: the same problem (as above) exists when discussing loss of sensitivity. Although this claim comes with simulated data, still the dimensionality is low (10 regions) and there is no model comparison result for different number of HMM hidden states and different number of PCA components. Accuracy of the state sequence through time is not adequate support for superiority of one method over another, when the correct number of latent states have been provided and no model evidence is reported. And even these accuracy values have not been statistically compared. Please reconsider these simulations.*

I cannot agree with the Reviewer on this point. For the reasons just exposed, an approximation of the model evidence (e.g. the free energy) is not the best manner to quantify the accuracy of an HMM representation here, especially when the ground truth is available. This figure was intended simply as an illustration of the potential loss of sensitivity when discarding PCA components that contain valid time-varying FC information. As before, this issue is a mere mathematical fact, not specific to a given data set. Since the Figure is intended as a demonstration of a problem that occurs under a particular condition (i.e. low-order, discarded PCA components carrying information), I believe that a grid search over PCA components and number of states would only cloud the main point being made by this Figure.

Regarding statistical testing, I have now included the results of running permutation testing. The differences are significant, as it is now indicated in the legend of the figure: “Permutation-based statistical testing revealed that HMM-PCA was significantly more accurate than the alternative approaches in all cases (p<0.001)”

Nevertheless, I agree with the Referee in that the paper would benefit for a more agnostic experiment. For this reason, I have added a new section in Results, with higher-dimensional data, a proper grid search over parameters, and including a report of the free energy. Please see Section 3.2.

*Page 8, section 3: Same problem. Maximum fractional occupancy and behavioral relevance can not replace model evidence. And please note that the behavioral relevance is barely different from zero. Perhaps if you compute the effect size alongside the p-value it becomes clearer that this may not be a very strong argument in favor of your model.*

Following on the previous point, I cannot agree with the Reviewer here. In typical neuroscience applications, it is much more preferrable to have a useful, interpretable model than a very accurate one. More importantly, because the ultimate goal is to understand the relation between brain mechanisms and behaviour, it is not very meaningful to have a computational model that bears no relation to behaviour –in the form of overt action or, else, as subject traits. Note that the reported small magnitude of the behavioural (cross-validated) explained variance is common in the field [33,34], while still being considered scientifically relevant if they are statistically significant [4], as it is the case here. I have now commented on the modest accuracies:

“In accordance with previous work [33,34] the accuracies in predicting HCP traits using FC features are relatively modest, with the average explained variance being lowered by traits that are particularly hard to predict (e.g. the personality traits). The average is, however, significantly higher than zero. Note that the grey lines connecting each trait's prediction between the two models reflect that the two models tend to perform better on the same traits.”

However please note that there was an error in the bars in the previous version, and the average accuracy (the bars) is actually lower than reported before for both models, although the dots were fine. As noted, this low average accuracy is in part due to the fact that some of the traits (for example those related to personality) are hard to predict [34].

Nonwithstanding, I still agree that reporting the model evidence (or free energy) is interesting and a good practice. However in this case, the free energy is unfortunately not very informative. This is because, since the HCP contains a large amount of data, the free energy’s likelihood term dominates the complexity term, favouring more and more complex solutions. Although quantitavely good in terms of accuracy, solutions with a very large number of states make fragmented models with scattered states that are not shared across subjects, and therefore are not reproducible. Since a large amount of the applications of the HMM to fMRI are seeking to quantify common patterns of covariation in FC across subjects, the fractional occupancy is a indeed a more useful measure in practice for this particular application.

As observed in Figure 2 of the Appendix, for all approaches the free energy favours solutions with more states and more principal components. Note that HMM-Gaussian and HMM-PCA are not directly comparable in terms of free energy, since HMM-Gaussian considers the principal components as observed data, whereas for HMM-PCA the principal components are Gaussian-distributed latent variables. This free-energy-based comparison has been referenced in the main text (start of the section and Fig 5 caption)

[34] U. Pervaiz, D. Vidaurre, M.W. Woolrich and S.M. Smith (2020). Optimising network modelling methods for fMRI. NeuroImage 211, 116604.

[33] R. Kong, J. Li, C. Orban, M.R. Sabuncu, H. Liu, A. Schaefer, N. Sun, X.N. Zuo, A.J. Holmes, S.B. Eickhoff and B.T. Yeo (2019) Spatial topography of individual-specific cortical networks predicts human cognition, personality, and emotion. Cerebral Cortex 29, 2533–2551.

[4] S.M. Smith, T.E. Nichols, D. Vidaurre, A.M. Winkler, T.E.J. Behrens, M.F. Glasser, K. Ugurbil, D.M. Barch, D.C. Van Essen and K.L. Miller (2015). A positive-negative mode of population covariation links brain connectivity, demographics and behaviour. *Nature Neuroscience* **18**, 1565-1567.

[19] C. Bishop. Pattern Recognition and Machine Learning. Springer 2006.

*This model has great potential and it deserves to be presented and motivated accordingly. Best of luck.*

Thanks. The Reviewer’s thoughtful comments have enriched the manuscript a lot, for which I am very grateful.

**Reviewer #2**:

*This article concerns the statistical difficulties in estimating time-varying functional connectivity in the brain from fMRI images where spatial dimensionality is high but the temporal windows for the time-resolved networks are small. The authors show that conventional time-averaged PCA methods can exhibit bias towards the least relevant components because all are given equal weight. They propose a hybrid method which uses (i) PCA to reduce the data at each time sample, and (ii) HMM to identify changes in the state of the FC network. The proposed method is tested on simulated and real fMRI data.

The paper is very well written overall. I especially appreciated the Introduction as I am not familiar with these statistical methods. Consequently my review is rather general in nature. I garnered that the proposed method improved the statistical result by using a single-step procedure to simultaneously reduce the data and estimate the functional connectivity at each state. However it was not clear to me how the algorithm worked. Specifically, I did not understand where the K different state-specific covariance matrices originated. Do they come out of the analysis?  Is the number K something that must be chosen beforehand? Perhaps the author can clarify these basic issues for readers who are not familiar with Mix-PCA.*

I appreciate the Reviewer’s positive comments, and I agree that the description of the method was somewhat technical. To make it more accessible, I have introduced a new paragraph to clarify these questions, where I explain from a higher-level perpective how the HMM algorithm works, including how the parameters are estimated and which hyperparameters (e.g. the number of states) must be specified a priori by the user:

“Both the HMM and the mixture model necessitate the number of states $K$ to be pre-specified. Although there exist extensions of the models where the number of states is directly inferred from the data (referred to as infinite HMM (Beal et al. 2002) or infinite mixture model (Rasmussen 1999), respectively), their parameter estimation is computationally demanding when we have large amounts of data and, in practice, they yield similar results to the standard models (Nielsen et al 2018). Given a specification of $K$, the estimation of the HMM or mixture model is here done through a process called variational inference (Bishop 2006; Vidaurre et al 2018), which, starting from a random initialisation of the state probabilities $\gamma_{tk}$, alternates the estimation of the state parameters ($\boldsymbol\Sigma_k$), the transition probability matrix and $\gamma_{tk}$ until convergence.”

*The layout of the manuscript could be improved. It is not clear where the Methods end and the Results begin. Methods sections 2.3 onwards would be better placed under Results.*

Following the Reviewer advice, I have placed the “Limitations of the two-step approaches” section now under Results.

*The results themselves are well-presented and I have no issues there.

Section 2.3.1 provides a good explanation of why the lower-order components in time-averaged PCA introduce biases. The third paragraph "When we apply PCA..." summarizes it particularly well. Section 2.3.2 provides nice results for the better sensitivity of the HMM-PCA method.*

I thank the Reviewer for her/his kind remarks.

*Section 3 compares the methods on real fMRI data and finds better results for the proposed method. The author admits (page 9 top) that is difficult to interpret the results in the absence of a ground truth in real fMRI data. Might it be possible to use a generative model of fMRI to test the algorithms in a scenario where the ground truth is known? Perhaps the author can comment on this in the Discussion.*

Following the Reviewer suggestion as well as the comments from Reviewer #1, I have designed a new experiment for which the ground truth is known. Although this cannot be taken as a completely realistic scenario (i.e. state transitions were not modelled based on biophysical considerations), the data was generated to replicate some of the features of the HCP. This is now in the new Section 3.3. Of course, more realistic (biophysical) simulations could be performed, but that would be slightly out of scope. I have commented on this in the Discussion: “A more complete description of which data sets would be more or less affected by these issues would possibly necessitate the use of simulations that are more biophysically realistic (Ritter et al. 2013)”

*Upon finishing the paper, I felt that it could have done more to make the proposed method available to the reader as a software package. At very least I encourage the author to make his existing code available as a demonstration. Otherwise the reader is obliged to transform the mathematical equations into their own software, which can be risky and time-consuming.*

I apologise for omitting this in the previous draft; that was not intentional. A toolbox including the four models (and others) was already online on a Github repository (<https://github.com/OHBA-analysis/HMM-MAR>). I now referenced all resources in the paper; see the Code Availability statement at the end of the paper.
 *Section 2.1, second paragraph. "... as zero mean Gaussian distributions."
Gaussian distributions of what? I didn't understand this statement.*

I have clarified this now in the text as follows:

“Within this general framework, we can define the states as covariance matrices \Sigma_k. In this case, the states are characterised as Wishart distributions or, equivalently, as zero-mean Gaussian distributions (Vidaurre 2021). This means that, during a given period of time when the $k$-th state is active, the data is considered to be generated according to the distribution

*Section 2.1, third paragraph. "... transient departures from orthogonality." I was confused what you meant by this. Do you mean that the principle components of one state differ from those of the next state? Can you please clarify?*

I apologise that this was not sufficiently clear. I have now rephrased as:

“Note that, across the entire PCA-reduced data set, $Y$, the $p$ PCs are by construction orthogonal (i.e. the correlation between the columns of Y is zero) . However, there might be periods in the data during which the time series are temporarily not orthogonal, such that the time series are temporarily correlated (or negatively correlated) across brain regions. Any transient departure from (the time-averaged) orthogonality, encoded by the HMM parameters $\gamma_{tk}$ and $\boldsymbol\Sigma_k$, can be regarded as an FC modulation.”

*Section 2.2, first paragraph. (1) Typo: "Various [of the] elements [of] this model are ...."*

This is fixed now. Thanks.

*(2) How is K determined? Please elaborate.*

As indicated above, K has to be chosen a priori by the user. I have included some text in Section 2.1 indicating this: “Both the HMM and the mixture model necessitate the number of states $K$ to be pre-specified”. This choice might obey to practical reasons or be driven by the purpose of the analysis.

*Section 2.2, second paragraph. "the Mix-PCA model uses K different PCA projections" Again, I wasn't clear to me where these K different projections came from.*

I have now rephrased to:

“To model the data, the Mix-PCA model uses, as states, $K$ different PCA projections, $W_k$, and their corresponding noise variance estimations $\sigma_k^2$, which are estimated from the data together with the state occupancies.”

*Section 2.2, second paragraph. "... using the EM algorithm”. What does "EM" mean? Perhaps I missed it but I don't think it was ever explained.*

I have now spelled out the acronym (expectation-maximisation), together with the appropriate references [26,27]. A Bayesian version of the EM algorithm (variational EM) is what I have used to estimate the parameters of the different models, for which there is some detail in the rest of Section 2.2. I hope it reads clearer now.

Section 2.2, third paragraph.  "...using the forward-backward equations ....". What are the 'forward-backward' equations? Are these a standard part of the Mix-PCA method?

I apologise for the confusion. The forward-backward equations are a standard part of the HMM estimation. I have clarified this in the text now (“As it standard practice…”).

*Section 3, Title: 'Results on real fMRI data’. This title is a little misleading as it suggest that the previous results (section2) are not based on real fMRI data. Whereas Fig 2 is based on real fMRI data, albeit manipulated data. Likewise for Fig 3, Scenario 2.*

To clarify this, I have included in the new Section 3.2 the paragraph: “In the previous section, I have used examples to illustrate the limitations of PCA when used in combination with the HMM or similar models. I now explore the comparative performance of the different models in higher-dimensional, more realistic data. Although these data are synthetic, I used aspects of real HCP data to perform the simulations;”

**Reviewer #3**

*In this paper the author has suggested a new approach in the HMM family that tries to combine PCA with HMM in a more unified way. The aim of this approach is to remedy the issue of high dimensionality of fMRI data. I find the method quite interesting and can think of many instances where such an approach would be quite useful. My major issue with this paper is that it seems a little rushed (for the lack of better term) in both introduction and discussion. I think if the authors add some more information to both introduction and discussion, the paper would be improved greatly.*

I thank the Reviewer for taking the time to revise the paper and for his useful comments: they have definitely helped to improve the paper.

*1. First and foremost, I do not find the title of the paper representing the core of this paper. This paper does not discuss the challenge of finding spontaneous changes in functional connectivity” a lot. Instead, a new approach is proposed that might help solve some of those “challenges”. When I read the title, I thought this paper is a review/commentary paper.*

I definitely agree with the Reviewer on this, so I have changed the title to: “Fusing dimensionality-reduction and time-varying functional connectivity estimation in a single model”

*2. I also find the introduction overly short. The authors have only given sliding window Pearson correlation (SWPC) as a different category of methods (the rest are all HMM). First although SWPC is one the most know method in this field, there are some more recent connectivity estimation that have been proposed recently. For example, shared trajectory by (Faghiri et al. 2020) or instantaneous phase synchrony (Pedersen et al. 2018) and even Multiplication of Temporal Derivatives (Shine et al. 2015). As these are more instantaneous estimators (compared to SWPC where the window size makes sure the estimand are not instantaneous) I find them more relevant to the current work and therefore should probably be cited too. Second, I don’t think these estimators should be compared to HMM at all. As HMM tries to directly estimates the FC states whereas these estimators aim to estimate connectivity and an additional step is usually required to estimate FC states from these connectivity time series. For example, one can use kmeans to estimate the FC states from connectivity time series (Allen et al. 2014). Right now if someone reads the introduction it seems you are comparing SWPC with HMM family which is not quite accurate.*

Thanks for the references. I have now expanded the Introduction in this regard and cited these papers. About the second point, I have now included the following sentence to make the connection with the HMM more natural and less confusing:

“[One possible strategy to quantify time-varying FC is the use of sliding-windows, where some measure of FC such as Pearson's correlation is computed across regions for each window in the data,] typically followed by the application of a clustering algorithm to extract patterns across windows” ,

*3. In the “Loss of sensitivity” section the author states “for example, this would correspond to a data set with sampling rate or TR≈1.1s, and 15min worth of data per session.” I do not understand how the authors has calculated the TR. Based on what I understand no sampling/resampling has been done to get a sampling rate value. This simulation can be from any other TR value too. And why is this information is given at all? The authors do not use it any other place to derive any kind of result.*

The Reviewer is right in that this sentence might read somewhat confusing. This was indeed not a result, and this value holds no relation with how the data were generated. It was just intended as illustrative: for that matter, I could have equally said “TR=2.2s and 30min worth of data”. To avoid further confusion, I have removed this phrase.

*4. Is it possible that the stability of the other HMM variants (gaussian HMM) is lower compared to the HMM PCA? i.e. can gaussian HMM predict better if it is run more than 5 times?*

That is an interesting point. Indeed, the stability of the HMM-Gauss is lower, as is now reflected in the new panel E of Figure 5. However, the predictions are still significantly better for the HMM-PCA variety when the prediction was done run per run (i.e. without pooling across runs and therefore without being able to leverage this additional variability). This rules out the explanation that it is this variability what is causing the differences.

Also please note that there was an error in the bars in the previous version, and the average accuracy is actually lower than reported before for both models.

*5. I do not see any mention of pre-processing of the fMRI data. Even if the authors have not done this at least a short summary would be needed (or a citation of where to find that information).*

Given that the HCP preprocessing is well-known and can be found in multitude of references, I preferred not to dwell too much on it. I have now included a brief remark about the preprocessing at the beginning of Section 3, citing an appropriate reference. Some more details about the parcellation, the TR, the acquisition time, and the number of subjects can be found at the beginning of Section 3.3.

*6. I find the discussion of this paper quite underwhelming. I think the author need to expand this part too. for example, a little more on the limitation of the proposed method? I see that the authors mention that their method is sensitive to the temporal ordering of the time series (which is true) but it has a limit. If I am understanding it correct it only care about temporal ordering for one time point. This can be considered a limitation as I believe this method would be unable to see slower changes. This point to another limitation of this study. The proposed method only find hard changes in the state and not the more fuzzy changes (at least the simulation is designed like that).*

Following the Reviewer’s suggestion and the other Reviewers’ comments, I have now expanded the Discussion including arguments about how to interpret this type of models and what makes a model “better” than another.

About the limitation of having an order-one Markovian assumption, in reality that’s only in terms of the conditional dependencies, which are now shown in the Figure 1 of the Appendix. That is, if we knew what state was active just before, the current state is independent of the previous time points. But in reality we do not know this, so the marginal distribution does depend on more than one time points in the past.

About the second limitation, the Reviewer is right: the exclusivity assumption of the HMM precludes modelling overlapping states, but probabilistically it can be fuzzily modelled. I have now briefly pointed at these limitations in the last paragraph of the Discussion, but I chose not to elaborate much on these points since these are not limitations of the HMM-PCA approach in particular, but of the HMM framework in general. In the text now: “Other limitations, such as the incapacity to model long-term temporal dependencies or the fact that the model does not allow for overlapping states, are however not specific of HMM-PCA but of the HMM framework more generally.”
