# Supplementary material for: A new model for simultaneous dimensionality reduction and time-varying functional connectivity estimation
Source: PLoS Comput Biol. 2021 Apr 16;17(4):e1008580. doi: 10.1371/journal.pcbi.1008580 (PMC8081334; doi:10.1371/journal.pcbi.1008580)
Supplement: Attachment — Submitted filename: Review2.docx [file pcbi.1008580.s003.docx]

*Reviewer #1: I appreciate that the manuscript has been improved with this revision. Performing more simulations, showing graphical models, computing model evidence, plus a number of other clarifications have all been valuable additions. I agree that HMM-PPCA is a potentially useful addition to the field of dynamic FC analysis.
However, there are still fundamental issues that we disagree about. In the following, I will elaborate on these points.*

I thank the Reviewer for taking the time again to review my paper. In summary, in this new version I have:

- Switched to cross-validated likelihood because of the limitations of the free energy, which I discuss below in detail.
- Run the behavioural predictions across a range of hyperparameters (no. of states and no. of principal components), as reflected in the new Figure 6, performing permutation-based statistical testing for each combination of hyperparameters and across all combinations.
- Discussed and hopefully clarified what I mean by distortion in the context of the HMM-Gaussian applied to PCA time series.
- Implemented other smaller changes suggested by the Reviewer.

Please find detailed responses below.

*• Introduction/motivation

To start with, please remember to cite the HMM-mixture-of-Bayesian-PCA model of Mauricio Alvarez and Ricardo Henao (2007) as prior work. Actually your HMM-PPCA model seems like a special case of their formulation.*

Thanks for the pointer, I have included this reference now in the first paragraph of Section 2.2.

From an organization point of view, the motivation section (called limitations of two-step approach) should go under Introduction, and not Results.

This is a sensible suggestion, but please note that this was how the paper was organised in the original version. At the suggestion of another Reviewer, this part was moved down to Results. In the end, I think the organisation will depend on the structure of the journal, so I will be happy to follow the Editor’s recommendation on that matter —if the paper gets published. However, the description of HMM-PCA in Methods should go before this, since, in the current version, HMM-PCA is mentioned here. For now, in this version I have made it a standalone section just before Results. About the actual content, please see next.

But more importantly, I don’t think the motivation content is appropriate.

*Specifically, I still can not sympathize with the distortion story of PCA, used to motivate your model. You are saying that two-step PCA+HMM produces results different from HMM on raw data, when many PCs are included. Lets forget for now that you showed us this distortion is about 1% for 84% explained variance. I repeat what I mentioned in the previous review: I don’t understand why anyone would expect PCA+HMM results to be identical to HMM. PCA is a data transformation/reduction technique. Of course it will discard certain details to facilitate discovery of the underlying structure in the data. This is not distortion, it’s the natural compromise of data reduction.*

I am sorry if that was not explained with enough clarity.

Please note that these are two different properties of PCA. One is that PCA loses information. As the Reviewer notes, and I completely agree, this is not a distortion, but the natural compromise of data reduction. The other property, and the one discussed here, is a change in the estimation above and beyond the loss of information –in particular when there is no loss of information as it is the case in Figure 3 (where all the PCs are retained).

As a counterexample, PCA is invariant to least squares regression: one can apply a PCA projection with no loss of information, regressing the PCs on a target variable, and we will have exactly the same prediction and explained variance than with the original data (up to the priors). Unfortunately, that is not the case of the HMM. This can be explained more clearly as follows:

When we use PCA, the eigenvalues contain information of the decomposition: i.e. if we randomly rescale the PCA scores we don’t have the same data anymore. HMM+Gauss, however, do not use the eigenvalues, as the scale of the eigenvectors is compensated by the diagonal of the covariance matrix (up to the priors; so for the sake of the argument please consider this is maximum likelihood). That is, we can completely change these scalings or eigenvalues, and the HMM would produce nearly the same solution (again, up to the priors). This is now shown in Figure 3A. From this, it follows that, by ignoring the eigenvalues, HMM-Gauss loses information. I refer to this as a distortion. Critically, PCA will always change the HMM estimation to a smaller or a larger extent **besides** the loss of information.

This is now reflected in the text as:

“I now discussed an intrinsic bias that will manifest regardless of how the time-varying FC information distributes across the PCs, and that will occur above and beyond the natural loss of information of PCA….”

“…A PCA decomposition has information in both the eigenvectors and the eigenvalues, but the eigenvalues are ignored in the HMM estimation, therefore changing the HMM estimation with regard to what would be obtained in the original data.”

Additional empirical evidence has been added in Panel C; see also the response to the next question below. Please note that Figure 3A already showed this empirically, even if it is a toy example. About the small amount of distortion of Figure 3B, this stood in stark contrast to Figure 3A, where the effect is clearly much larger (see first vs last row). I have now realised that this is because the simulation was performed differently for the two panels. In this version, I have made the simulation of panel B just like panel A, and the effect has increased considerably. Specifically, this is now done such that all models start from the same initialisation; before panel B was done such that one model was initialised with the other model’s final solution, which, perhaps, might have been leading to suboptimal local minima.

*If you still see this as an important problem, have you at least shown that HMM-PPCA solves it? Have you shown that HMM-PPCA results are very similar to HMM on raw data? I didn’t find any such results. When you stress an issue as the motivation of your work, the reader is waiting to see how you have resolved this issue.*

This is a good question. But please note that this experiment is not possible by the own definition of the models. That is, one can show that applying PCA changes the HMM-Gauss estimation even if all variance is retained (Figure 3), but one cannot show an equivalent thing with HMM-PCA because there is no PCA-less version of HMM-PCA. Comparing HMM-PCA with a PCA-less HMM-Gauss estimation is also ill-defined because these are different models with different numbers of parameters (in particular, one implies dimensionality reduction whereas the other does not). However, it follows from first principles that HMM-PCA does not have this issue, as is now pointed out:

“It follows the question of whether HMM-PCA is able to solve this problem. It can be shown theoretically that this is the case, because, as discussed, the issue has to do with having state-specific error variance parameters, while in our HMM-PCA formulation the error covariance matrix (and therefore error variance) is common to all states.”

Although it is hard to show direct empirical evidence of this, I now show indirect evidence by reversing the argument. The new Figure 3C panel and the following text have been added to the manuscript:

“Empirically, we can manipulate the data by randomising their eigenvalues (i.e. by multiplying the ordinary PCA weights by a random number). Since this is changing the nature of the data, the HMM estimation, if correct, should also change to reflect the manipulation. If it does not change, that signals a problem. Figure 3C shows the correlation between the HMM estimation (i.e. between the state time courses) before and after performing this manipulation in the data, for both HMM-Gaussian and HMM-PCA. As shown, HMM-PCA reflects that the data have changed by changing its estimates, while HMM-Gaussian is mostly unaffected. Although this can only be considered as indirect evidence, it adds further evidence to the issue discussed in this section.”

*I understand you need to motivate the model. Why not use all the motivations in the mixPPCA/mixFA literature? For example: the benefits of local linear sub-models, having fewer parameters to estimate than a full-covariance Gaussian mixture model, not having to impose axis aligned covariance matrices to reduce the number of parameters, all the advantages of probabilistic PCA over classic PCA, the temporal structure modeled with HMM, etc. I am sure you know the following references, so you can easily get inspirations from their introductions: Bishop and Tipping (1999), Bishop and Winn (2000), Ghahramani and Beal (1999).* *If you have been thinking that global PCA prior to HMM is not as efficient as local PCA (using mix-PPCA or your extension as HMM-PPCA) in capturing and preserving local structure, I totally agree. But this is not what you have explained for the reader.*

I totally agree with the Reviewer about the advantages of HMM-PCA over (P)PCA as well as the other arguments. However, I would like to emphasise the target audience of this paper: the neuroscience community; as well the specific goal I am addressing: the quantification of time-varying FC. The benefits of HMM-PCA as opposed to PPCA are clear and have been, in general terms, discussed beforehand in the literature. Although this and the other points could easily be made here too, the motivation of this paper must be with respect to a time-varying FC method –in this case HMM-Gaussian. Please note that the point of “*that global PCA prior to HMM is not as efficient as local PCA (using mix-PPCA or your extension as HMM-PPCA) in capturing and preserving local structure*” has already been discussed and is the object of the other motivating subsection: “Loss of sensitivity”. Since this section appears to be more intuitive, I have promoted it to go first (so Figure 2 and 3 are now swapped).

o Simulation Design

*Simulations should be designed to showcase the important aspects of a proposed model. In the new high-dimensional simulations of the manuscript, the (effective) dimensionality of the data, per component/state, is not specified. This is important because you need to show that HMM-PPCA can recover both the number of HMM latent states and the intrinsic dimensionality of each state-specific PPCA. Actually your lower-dimensional simulations had this aspect encoded in the number of retained eigenvectors of the covariance, but the new simulations miss this point. When we don’t know the dimensionality of the principal subspace in the simulations, we can not judge the model inversion and comparison results.*

As it is now explicitly specified in the text, the effective dimensionality of the data per state (i.e. regarding to the ground truth) is always 100 x 99 / 2 = 4950 parameters, which is the number of off-diagonal elements in the full-rank covariance matrices. This is so because I did not truncate the corresponding eigendecompositions and used all eigenvectors. In the HMM-PCA estimations, every state has 100 x *p* parameters, with *p* within {5, 10, 20, 30, 40}. In the HMM-Gaussian, this is p x (p-1) / 2. All of these numbers are below 4950. This is now reflected in the bottom panel in Figure 4, where I show accuracy versus number of parameters.

*o Coupled update equations

(Expected) complete data log-likelihood (Eq. 4) should be written for the whole HMM-PPCA model (not per state), in which case the EM update equations of PPCA and HMM become coupled. Intuitively, this is because the likelihood term (used to update the HMM state) now depends on PPCA posteriors as well. This is an important point to show, as you are offering a unified model. Check out the following references for similar derivations of coupled update equations:
- Bishop and Tipping's (1999) mix-PPCA (Appendix C, Eq. 73)
- Bishop and Winn’s (2000) variational treatment of mix-BPCA (Section 3.1)
- Alvarez and Henao’s (2007) derivations for the HMM-mix-BPCA model (Appendix B)*

Thanks – I have added the complete data log-likelihood equation now (Eq. 4).

*o Latent space identification

My concerns here can be best summarized as a question: How does the author intend to identify the optimal number of latent states for HMM and PPCA on empirical data? There is no answer in the manuscript.*

In the new version, I use cross-validated likelihood as a measure to automatically select the complexity of the model. As I now mention in the text:

“The cross-validated likelihood, used as a quantitative way to assess the models and perform model selection, is sometimes preferred to other methods based on penalised likelihood when the model assumptions are too far from the true generating distribution of the data as it is the case with brain data.”

Please see references [1,2] on that matter.

[1] P. Smyth (2000). Model selection for probabilistic clustering using cross-validated likelihood. Statistics and Computing 10.

[2] Celeux and Durand (2008). Selecting hidden Markov model state number with cross-validated likelihood. Computational Statistics 23.

Therefore, now I present the cross-validation likelihood for both the synthetic simulations in the top panels of Figure 4, instead of the free energy. This is now discussed in the text as:

“Figure 4 shows the results of the analysis.The top panels reflect the cross-validated likelihood. In all models, the cross-validated likelihood is able to estimate that at least 6 states are required, but it can hardly distinguish between 6 and higher values of K, suggesting that for 7 and 8 states the amount of model overfitting is negligible. For HMM-PCA and Mix-PCA, the cross-validated likelihood favours solutions with larger numbers of PCs. For HMM-Gaussian (which input data is Y), we cannot straightforwardly compare different numbers of PCs, and neither can it be compared to HMM-PCA (which input data is D), since the likelihood is computed using different data in each case. The middle panels show the error in predicting the ground state time courses, and the bottom panels show that same information as a function of the number of parameters. HMM-Gaussian and HMM-PCA have similar accuracies despite the fact that the ground-truth generating distribution is Gaussian. HMM-PCA outperformed Mix-PCA, specially in the harder ε=0.1 case (right panels), which highlights the importance of the HMM temporal regularisation.”

*Also note that the dimensionality of the principal subspace can differ across states/components in the HMM-PPCA model. In your application this means that different functional states can express their intrinsic complexities through the dimensionality of their respective principal subspaces. So, on the one hand, optimizing the latent space is essential for model fitting on a given dataset. On the other hand, this step is important for between-group analysis, e.g. between a normal and patient group. Once the model has been fitted to each dataset separately, one may find out that the functional components from one group fit into lower dimensional subspaces, or may need fewer HMM states to be explained, than the other group, which can be nicely interpreted. If latent variable dimensionalities have not been optimized on each dataset, such between-group analysis would not be feasible.*

In this case, we are not imposing an ARD/sparse prior on the observation models, so the effective dimensionality will unfortunately not be adaptively tuned across states or components – in other words, all states will have the same number of parameters for a given choice of *K* and *p*. In future developments, I will consider imposing appropriate priors on *W*, a strategy that indeed worked reasonably well for our group in the past (Vidaurre et al., 2016; Harrison et al. 2015). I now mention this perspective in the limitations paragraph of the Discussion:

“An efficient application of prior distributions on the PCA weights W_k, for example promoting sparsity, is a promising avenue to help in this direction.”

*• Model comparison with free energy

Please note that what I call free energy (F) is the negative of free energy in physics, or ELBO in machine learning. There could be a number of reasons why free energy is not guiding your model comparison to optimize the latent space dimensionality and to compare competing models of different structure:*

For the reason explained above, I am not using the free energy in the current version of the paper, but see some comments below.

*1) I suspect that you have not computed free energy for the whole hierarchical model. I had a glance at your code, and F seems to pertain to the HMM part of the model only. I can’t see the complexity terms neither for the PPCA nor for the transition matrix. If that is indeed the case, it explains why F is not penalizing higher complexity and keeps encouraging more and more dimensions/states (similar to over-fitting of maximum likelihood schemes). You may want to have a look at the Appendix of Friston et al. (2015) to see how the free energy of a hierarchy is written in terms of the expected free energy of the lower level minus the complexity of the higher level.*

The computation of the free energy did include the complexity term for the transition matrix (line 84 in evalfreeenergy.m), but it is true that it didn’t for the observation model, since I did not impose a prior on the PCA weights’ distribution, and, therefore, the complexity term was here not well-defined.

*2) A second problem might be the way you are parametrizing the likelihood. I remember I mentioned in the previous review that taking out the ‘mean’ is not justified. That is because when you fix all the means to zero, it’s like trying to disentangle concentric ellipsoids. You may want to put the mean back and see whether the state cardinality shows itself.*

Please note that this cannot be the case in the simulations because the ground truth does not have a mean parameter.

In the real data scenarios, the Reviewer is right in that removing the mean can be suboptimal from a modelling point of view. However, please note that, as I discussed in the previous response letter, this choice was imposed by the scientific question that is the object of this paper: the existence of changes in functional connectivity above and beyond changes in amplitude.

*3) A third solution is adopting a fully Bayesian treatment (with prior over PPCA loadings). At least for PPCA dimensionality discovery this approach has proven very successful in the literature.*

I agree with the Reviewer on this point, and I will consider an extension of the model in this direction. As mentioned, I comment on this on the paragraph about limitations in the Discussion:

“An efficient application of prior distributions on the PCA weights W_k, for example encouraging sparsity, is a promising avenue to help in this direction.”

*• Replacements for model evidence

The author seems to propose fractional occupancy of states and behavioral relevance as model evaluation criteria. As I explained before, these can not replace model evidence. (As a side note: one can use other approximations to model evidence, other than F, or even use sampling to compute model evidence).*

*Speaking of fractional occupancy, I have seen in the literature that a balanced fractional occupancy of states is more probable in healthy brains, than pathologic ones. However, fractional occupancy is not the cost function for optimizing the parameters of your model. This sort of domain-specific information can be encoded as ‘prior’ belief, e.g. on the form of the transition matrix. As for behavioral relevance, only once the model optimization is over can one inspect relation to behavior. Comparing behavioral relevance on (structurally different) models with arbitrarily chosen parameters is not meaningful, to claim superiority of one model over another.*

Interpretation and quantitative accuracy are two different goals that do not necessarily have to coincide. In engineering applications (and research papers in machine learning), it is critical to have a quantitative model selection procedure based on, for example, model evidence or cross-validation. In basic neuroscience, the relation to behaviour of the models is however the ultimate purpose. For the basic research question that we are addressing in this paper, if the model has no relation to behaviour, then –even if a model fits the neural data better than any other– such model has no use. If we were addressing a predictive problem in applied neuroscience (e.g. anticipating epileptic seizures), I would be in full agreement with the Reviewer. I briefly discuss this question in the second paragraph of the Discussion.

Having said that, I agree with the Reviewer in that comparing behavioural relevance (and fractional occupancy) for a given configuration of parameters does not need to generalise for other configurations of parameters. To address this question, I have run the behavioural prediction over a grid of parameters, including the number of states and the number of PCs. The results are now shown in the new Figure 6. As discussed in the main text, I have used the non-parametric combination algorithm to integrate the different tests across the different configurations of parameters, showing that, in general (at least within this data set), HMM-PCA is superior in this sense to HMM-Gaussian.

Further, following the discussion with the Reviewer in this and the previous review, I now realise that the maximum fractional occupancy is not a rigorous method of comparison. This is because we now know from ongoing work that the amount of parameters in the model has a strong influence on state mixing (defined as how much diversity of states we have per subject, here quantified with the maximum fractional occupancy –panel C in Figure 5, in the previous version). For this reason, I have removed all references to this statistic in the current version of the paper.

The Reviewer also makes a good point that behavioural relevance is not a cost function of the model. But please note that this is not necessarily a bad thing. In fact, having this as a separate criterion from the cost function allows us to easily validate the model in a non-circular way, which would be compromised if we include this information as prior beliefs. Most importantly, I would like to reemphasise that behavioural relevance is often the most important from a scientific point of view.

*I think taking care of these points would go beyond a standard revision. I would encourage the author to go through the reviews, repeat some of the analyses, and resubmit a fresh version of this work. I still think the idea is great and should definitely be added to the literature soon. Best wishes.*

I very much appreciate the time taken by the Reviewer and their thorough revisions. I think this is very generous, and I feel that as a result the paper has improved a lot from the first version.
